# Determinants of clinical outcome in patients with moderate/severe Graves' orbitopathy undergoing treatment with parenteral glucocorticoids: a retrospective study

**DOI:** 10.3389/fendo.2024.1401155

**Published:** 2024-07-04

**Authors:** Rosario Le Moli, Adriano Naselli, Gabriele Costanzo, Tommaso Piticchio, Dario Tumino, Gabriella Pellegriti, Francesco Frasca, Antonino Belfiore

**Affiliations:** ^1^ Endocrinology Unit, Department of Clinical and Experimental Medicine, Garibaldi Nesima Hospital, University of Catania, Catania, Italy; ^2^ Department of Medicine and Surgery, University of Enna "Kore", Enna, Italy

**Keywords:** parenteral glucocorticoids, oxidative stress, Graves' orbitopathy phenotype, LDL - cholesterol, hypertension

## Abstract

**Background:**

Graves' orbitopathy (GO) occurs in approximately 25-40% of patients with Graves' disease (GD). High levels of anti-thyrotropin receptor antibodies (TRAbs), smoking habit, sex, older age, longer duration and amount of hyperthyroidism or hypothyroidism are well-recognized risk factors for the occurrence, severity and clinical course of GO. Oxidative stress (OX) has recently been shown to play a role in the pathogenesis of GO, and several clinical conditions related to OX have been investigated regarding the presentation and severity of GO.

**Aim:**

We aimed to evaluate the impact of clinical conditions related to oxidative stress on the outcome of intravenous glucocorticoid (ivGCs) therapy in a cohort of patients with active moderate to severe GO (AMS-GOs) treated at a single institution.

**Methods:**

We retrospectively studied a series of patients with AMS-GOs who were treated with ivGCs from January 2013 to May 2022. GO clinical evaluation was performed at baseline and at 6 (W6), 12 (W12) and 24 (W24) weeks after starting ivGCs by the seven-point clinical activity score (CAS) alone and by overall clinical criteria (CI) according to the European Group of Graves' Ophthalmopathy (EUGOGO). Total cholesterol and calculated LDL cholesterol (LDLc), triglyceride, body mass index (BMI), diabetes status, history of hypertension (HoH), smoking status, age and sex were used as covariates for the clinical outcome of GO to ivGCs.

**Results and conclusions:**

LDLc and HoH negatively and independently modulated the response of AMS-GOs to ivGCs. Notably, slightly elevated LDLc levels (> 130 mg/dl) reduced the response of orbital soft tissue to ivGCs, whereas more elevated LDLc levels (from 175 mg/dl to 190 mg/dl) and HoH were associated with poorer clinical response of eye motility and proptosis.

## Introduction

Graves' orbitopathy (GO) is an extrathyroidal manifestation of Graves' disease (GD) (1). GO occurs in approximately 25-40% of patients with GD, before, during, or after GD presentation, and presents with varying degrees of activity and severity (2). Smoking habit, sex, older age, high TRAbs level, and longer duration of hyperthyroidism or hypothyroidism are well-recognized risk factors for the occurrence and severity of GO (3). OX has recently been shown to play a role in the pathogenesis of GO, and several clinical conditions related to OX have been investigated regarding the presentation and severity of GO. Metabolic disorders are among the OX-related conditions (4–6). Indeed, type I diabetes mellitus (T1DM) is more common in patients with GO than in individuals in the normal population, and dysthyroid optic neuropathy (DON) is more common in patients with GO and T1DM and has a worse prognosis than in nondiabetic patients (5, 7). In GD patients with type II diabetes (T2DM), GO precedes the onset of hyperthyroidism more frequently than it does in control patients. GO severity was also significantly associated with T2DM lasting longer than five years (odds ratio = 4.9; p = 0.045) and with the presence of microvascular and macrovascular complications (odds ratio = 4.8; p = 0.048). GO severity is also associated with patient overweight (BMI > 26) (5). A recent cross-sectional study indicated that high levels of total cholesterol and LDLc are associated with GO presentation and activity (8). Accordingly, Stein et al. reported that statin therapy may reduce the incidence of GO in patients with GD (8, 9). Indeed, hypercholesterolemia can cause chronic inflammation *per se* or through oxidized LDLc (oxLDLc), while statins have a protective role owing to their anti-inflammatory and hypolipemic actions. Very elevated LDLc levels reduce the clinical response rate of GO to intravenous glucocorticoids (ivGCs) (6). Moreover, a recent phase II, randomized, open-label clinical trial revealed that atorvastatin improves the response of hypercholesterolaemic patients to GO in combination with ivGCs, regardless of blood cholesterol levels (10). No other data are currently available regarding the role of clinical conditions related to OX in the response to ivGCs in patients with moderate to severe active GO (AMS-GOs).

In this retrospective study, we evaluated the impact of clinical conditions related to OX on the outcome of ivGCs therapy in a cohort of patients with AMS-GOs treated at a single institution.

## Patients and methods

### Patients

GO clinical evaluation was performed at baseline and at 6 (W6), 12 (W12) and 24 (W24) weeks after starting ivGCs.

Inclusion criteria were: patients with GD and untreated active AMS-GO, stable thyroid function for almost one month. Exclusions criteria were: any previous treatment of the GO, 131 Iodine or thyroidectomy, CAS < 3, instable thyroid function or non-adherence to taking anti thyroid drugs, pregnancy, any other clinical condition where the use of ivGCs was not indicated.

Fifteen patients were excluded because had received 131 Iodine, 27 patients that underwent to thyroidectomy and 34 patients that received glucocorticoids or retrobulbar radiotherapy for GO before ivGCs were not included in the study. 160 patients with GD who were treated with ivGCs because AMS-GO at our institution from January 2013 to May 2022 were initially included in our database, 21 patients were excluded because analytical and/or demographic data were not complete. Therefore, a total of 139 patients with GD and AMS-GO diagnosed according to EUGOGO criteria were included in the study (11).

The same ophthalmologist performed the ophthalmologic assessment which consisted of recording palpebral aperture (in mm) in primary position, soft tissue involvement (according to EUGOGO atlas) (11), exophthalmos (in mm by Hertel exophthalmometer), involvement of extra ocular muscles (ductions measured in degrees; we graded ocular motility involvement as mild (grade A involvement: plus or minus 8 degree in duction), moderate (grade B involvement: plus or minus 16 degree in duction), severe (grade C involvement: improvement or deterioration of ocular motility more than 16 degree in duction); Bahn and Gorman diplopia score and visual acuity (in decimals using Snellen chart). Also, the 7-point Clinical Activity Score (CAS) was evaluated (12). GO was considered active when CAS was ≥ 3. Improvement (I) was defined as the change in two of the following outcome measures in at least one eye without deterioration in any of the same measures in both eyes: 1) improvement of the CAS by at least 2 points, 2) improvement of soft tissue involvement according to EUGOGO atlas, 3) improvement of proptosis of at least 2 mm, 4) improvement of the lid aperture of at least 3 mm and 5), improvement of diplopia, disappearance/change degree according to Gorman Score or amelioration of one grade of ocular motility. Not improvement (NI) was defined when above points worsened to the degree as described for improvement (e.g. deterioration of CAS by at least 2 points, etc.), or when DON occurred.

The following OX-related clinical conditions were selected as covariates with respect to the outcome evaluated by CI: total cholesterol and calculated LDLc, triglyceride, body mass index (BMI), diabetes, history of hypertension (HoH), smoking status, age, and sex.

### Pulse therapy

Corticosteroid treatment for GO consisted of ivGCs (Solumedrol; Pfizer, Karlsruhe, Germany) injections with a median cumulative dose of 49 mg/kg subdivided into 12 weekly infusions. Patients were asked to visit our endocrinology day hospital on the appointed day; an indwelling venous catheter was inserted into the antecubital vein between 8.30 and 9.30, and Solumedrol diluted in 250 ml of a 0.9% sodium chloride solution was administered at an infusion rate of 120 ml/h in the postabsorption state and in the lying position. Blood pressure; blood glucose; cholesterol, HDL, triglycerides, TSH, FT3, FT4, TRAb, GOT, GPT, GGT, bilirubine, creatinine, azotemia and blood count were evaluated before to and every 3 weeks after the starting of ivGCs infusion.

### Analytical methods

Serum hormones were measured by a microparticle enzyme immunoassay (Abbot AxSYM-MEIA) with interassay coefficients of variation of less than 10% over the analytical ranges of 1.7–46.0 pmol/L for FT3, 5.15–77.0 pmol/L for FT4, and 0.03–10.0 mU/L for TSH. The within-run and between-run precisions for the FT3, FT4, and TSH assays showed coefficients of variation <5%. TRAbs were measured by a III generation assay [SELco TRAbs Human, Dahlewitz/Berlino (Germany)]. Glycaemia, total cholesterol, high-density lipoprotein (HDL) cholesterol and triglyceride levels were evaluated by standard methods. We calculated LDLc values by the formula of Martin/Hopkins, which is reliable for a wide range of triglyceride values (13). The thyroid gland was evaluated by ultrasound, and the volume of each lobe was calculated with the following formula for ellipsoid volumes: Volume = length*width*depth*(p/6). The calculated thyroid volume was the sum of the volumes of the two lobes (14).

### Statistical analysis

Sample size computation was performed using G*Power 3.1.9.7. We estimated that to obtain a p value ≤ 0.05 with a statistical power of 0.8 a total of 54 patients would have needed with a low association with other covariates (R2 = 0.04), while 69 would have needed with a moderate association (R2 = 0.25). All the statistical analyses were performed with the SPSS package (IBM SPSS Statistics for Windows, Version 26.0. Armonk, NY: IBM Corp.). For descriptive analysis, continuous variables are expressed as medians (25th-75th percentiles); categorical variables are expressed as numbers and percentages.

The shapes of the distributions of each variable were evaluated by visual inspection of the population pyramid charts. Univariate analysis was performed by the Mann–Whitney U test or chi-square test where appropriate. After that, we performed multiple univariate regression analyses including the following covariates as OX conditions: total cholesterol and calculated LDLc, body mass index (BMI), fasting glycaemic values, history of hypertension (HOH), smoking, age and sex setting I or NI at W6, W12 and W24 as outcome variables. Early response (at W6) to ivGCs was also evaluated with respect to clinical outcome at the different time points. We then constructed a multivariate logistic regression model using the putative predictors selected by univariate analysis. Logistic regression analysis was carried out on the W6CI, W12CI and W24CI outcomes. Covariates were selected according to the results of the univariate analysis. The existence of a linear relationship between the logit of the outcomes of different variables and continuous predictive variables was verified by the Box-Tidwell procedure. A Bonferroni correction was applied by using all the terms in the model to evaluate the statistical significance of the differences. Multicollinearity was ruled out after evaluating the tolerance and variance inflation factor (VIF). The presence of outliers was verified by analyzing standardized residues and by reporting standard deviation values of ≥1.96 or ≤-1.96. All the patients in which the standardized residue concentration exceeded the aforementioned standard deviation thresholds were re-examined. The choice of inclusion or exclusion of these patients from the analysis was performed after evaluating whether these patients exerted an inadequate influence on the model by using the following statistics: Cook's distance, DFBeta and Leverage statistics. The adequacy of the models was tested through the method of maximum likelihood. The Wald test was applied to verify whether the coefficients differed from 0. Associations between each predictive variable and the outcome were quantified through odds ratios (ORs). The Nagelkerke pseudo-R2 value was used to evaluate the goodness of fit in logistic regression analysis by the variance proportion of the dependent variable determined by each single model (15–18). We finally provided a factorization of the CI (Factor a: soft tissue involvement, Factor b: Hertel measurements, Factor c: presence of diplopia, Factor d: eyelid aperture and Factor e: CAS) with the aim of comparing each single clinical parameter to selected variables based on the results of the univariate analysis. The chi-square test and Fisher's exact test were also used for this process. The continuous variables included in this multiparametric analysis were switched to categorical variables to establish cut-offs for each CI parameter through visual inspection of the graphs. The specific cut-offs were chosen with reference to the Adult Treatment Panel III standard levels for LDLc (19).

Moreover, only the patients who were starting from a condition of "potential for improvement" at the beginning of pulse therapy were included for each parameter. Therefore, patients whose physiological condition started for each individual parameter were excluded from the analysis. In this way, patients were not erroneously classified as "not improved".

## Results

The characteristics of the patients studied are depicted in **Table 1**.

**Table 1:** Baseline characteristics of the 139 patients of the study.

**Age** (years)	46 (34-56)
**Sex** (F/M)	40/99
**ivGCs** (mg/kg)	49 (36-65)
**BMI** (Kg/m^2^)	25 (16-31)
**TSH** (mU/L)	0.1 (0-1.4)
**FT3** (pg/ml)	3 (2.6-3.7)
**FT4** (ng/dl)	1 (0.9-1.3)
**TRAb** (U/L)	5.3 (2.3-18)
**CAS**	3 (3-4)
**Diplopia** (n,%)	28 (20.1)
**Thyroid function** (S. Hyper./Hyper./Eu.) (n.)	85/9/45
**GD duration** (months)	14 ± 10
**GO duration** (months)	10 ± 7
**Therapy (**n.) ATD/ATD+L-T4/L-T4/none	59/47/25/8
**Glycaemia** (mg/dl)	84 (87-103)
**Cholesterol** (mg/dl)	200 (176-237)
**LDLc** (mg/dl)	123 (101-159)
**Triglyceride** (mg/dl)	83 (64-138)
**HDLc** (mg/dl)	55 (47-62)
**Statins** (%)	8.6
**Diabetes** (%)	7.9
**HoH** (%)	24.5
**Smoke habit** (%)	49.6

ivGCs, intravenous glucocorticoids; BMI, body mass index; FT4, free tetraiodothyronine; FT3, free triiodothyronine; TSH, thyroid stimulating hormone; TRAbs, thyrotropin receptor binding immunoglobulins; S. Hyper., sub clinical hyperthyroidism; Eu., euthyroidism; HDLc, hight density lipoprotein cholesterol; LDLc, low density lipoprotein cholesterol; CAS, clinical activity score; HoH, hypertension history.

### Evaluation of outcomes at weeks 6, 12 and 24 according to the CAS

One hundred thirty-nine patients with AMS-GOs, 40 males and 99 females with a median age of 47 (range 36-55) years that received a median cumulative ivGCs dose of 49 (range 36-65) milligrams/kg body weight were included in the study (**Table 1**).

According to the CAS, 77.9% and 75.5% of all patients were classified as "improved" (I) at week 6 (W6) and W12, respectively. A total of 86 patients completed the follow-up at W24, and 67.4% of them were classified as I.

### Evaluation of outcomes at W6, W12 and W24 according to CI

According to the CI, 45.9% of patients were classified as I at W6, and 43.2% were classified as I at W12. Among the 86 patients who completed the follow-up at W24, 38.4% were classified as I (**Table 2**). According to the univariate analysis, LDLc was significantly elevated in the patients classified as 'nonimproved' (NI) compared with the I group (p= 0.003 at W6, p = 0.03 at W12 and p = 0.07 at W24; Mann–Whitney U test). Similarly, HoH and age were negatively associated with GO improvement (for HoH: p=0.07, p=0.01 and p=0.02 at W6, W12 and W24, respectively; chi-square test; for age: p=0.02, P=0.008 at W12 and W24, respectively; Mann–Whitney U test). Fasting glycaemia, smoking status, sex and BMI were not associated with GO improvement (**Tables 3**, **4**).

**Table 2:** Short, medium and long term clinical outcome to ivGCs according to CAS and CI.

Outcome	W6		W12		W24	
	CAS	CI	CAS	CI	CAS	CI
**I** (%)	77.9	45.9	75.5	43.2	67.4	38.4
**NI** (%)	22.1	54.1	24.5	56.8	32.6	61.6

W6, week six; W12, week twelve; W24, week twenty-four; CI, overall clinical criteria according to EUGOGO; I, improve; NI, non improved.

**Table 3:** Univariate analysis: *Man Withney - U test*.

	Outcome	W6	*P*	W12	*P*	W24	*P*
**Age** (Y)	I NI	46 (33-55) 49 (36-56)	*0.4*	44 (34-51) 52 (39-56)	*0.02*	38 (31-50) 50 (37-53)	*0.008*
**ivGCs** (mg/kg)	I NI	48 (37-59) 45 (36-65)	*0.8*	49 (38-63) 45 (35-67)	*0.6*	50 (38-65) 49 (36-68)	*0.9*
**BMI** w/(heigth)^2^	I NI	25 (23-29) 26 (23-31)	*0.4*	24 (22-29) 26 (21-31)	*0.9*	26 (23-31) 25 (21-28)	*0.2*
**FT4** (ng/dl)	I NI	1 (0.9-1.2) 1 (0.8-1.1)	*0.8*	1 (0.9-1.2) 1 (0.9-1.3)	*0.6*	1 (0.9-1.3) 1 (0.9-1.2)	*0.3*
**FT3** (pg/ml)	I NI	3 (2.6-3.5) 3 (2.6-3.7)	*0.2*	3 (2.5-3.4) 3 (2.7-3.7)	*0.1*	3 (2.6-3.6) 3 (2.6-3.7)	*0.9*
**TSH** (mU/l)	I NI	0.2 (0-1.1) 0.1 (0-1.4)	*0.8*	0.2 (0-1.1) 0.1 (0-1.4)	*0.5*	0.2 (0-1.3) 0.1 (0-2.1)	*0.9*
**TRAbs** (IU/l)	I NI	3 (1.3-6.2) 6 (2.1-17)	*0.1*	3 (1.4-8.4) 6 (3.4-18)	*0.1*	3.6 (2.6-11.8) 5.2 (1.9-21.4)	*0.7*
**Glyc.** (mg/dl)	I NI	94 (87-103) 94 (87-101)	*0.7*	95 (86-104) 94 (87-101)	*0.5*	95 (89-104) 94 (87-101)	*0.3*
**Chol.** (mg/dl)	I NI	190 (166-214) 202 (181-229)	*0.04*	192 (165-216) 202 (180-237)	*0.05*	190 (169-212) 196(178-235)	*0.2*
**HDLc** (mg/dl)	I NI	56 (49-66) 53 (47-60)	*0.1*	53 (48-62) 55 (47-60)	*0.8*	52 (45-62) 55 (49-62)	*0.4*
**Triglyc.** (mg/dl)	I NI	76 (57-111) 89 (68-138)	*0.03*	80 (60-127) 86 (66-131)	*0.3*	78 (60-126) 86 (69-131)	*0.3*
**LDLc** (mg/dl)	I NI	109 (91-130) 126 (107-152)	*0.003*	119 (94-134) 126 (107-159)	*0.03*	115 (96-130) 125 (105-159)	*0.07*

**Table 4:** Univariate analysis: *Chi square Test*.

	Outcome	W6	P	W12	P	W24	P
**Sex** (F/M)%	I NI	41/44 59/56	0.7	43/40 57/60	0.8	57/32 43/68	0.07
**Statins** (Y/N)%	I NI	60/40 40/60	0.3	60/39 40/61	0.1	40/38 60/62	0.9
**HoH** (Y/N)%	I NI	21/46 79/54	0.07	13/47 87/53	0.01	12/44 88/56	0.02
**Diabetes** (Y/N)%	I NI	75/40 25/60	0.1	60/40 40/60	0.3	20/39 80/61	0.6
**Smoke** (Y/N)%	I NI	49/34 51/66	0.1	44/36 56/64	0.3	42/34 58/66	0.5

Next, we performed multiple univariate regression analyses. Patients with GO that improved at W6 were 8 and 7 times more likely to be classified as I at W12 and at W24, respectively (OR 8.1, 95% CI 2.8-23.0, p<0.001 and OR 7.0, 95% CI 2.5-19.7, p <0.001), I vs. NI). Furthermore, total cholesterol and LDLc were significantly associated with no improvement (p=0.03 at W6 and P=0.04 at W12 for total cholesterol; p=0.003 at W6; p=0.01 at W12; and p=0.04 at W24 for LDLc). HoH and age were associated with nonimprovement at W12 (p= 0.003 and p= 0.02, respectively) and W24 (p= 0.03 and p= 0.01) (**Table 5**).

**Table 5:** Multiple univariate regression analyses.

	(P) W6 outcome	(P) W12 outcome	(P) W24 outcome
**W6 outcome**	**——**	<0.001	<0.001
**Total Cholesterol**	0.03	0.04	0.12
**LDLc**	0.003	0.01	0.04
**BMI**	0.4	0.9	0.3
**Fasting Glycemia**	0.4	0.3	0.3
**HoH**	0.1	0.003	0.03
**Smoking**	0.9	0.9	0.4
**Age**	0.5	0.02	0.01
**Sex**	0.9	0.7	0.06

We then constructed a multivariate model aimed at predicting clinical GO outcomes at W6 and W12. This model was significant, χ^2^(3)=15.094, p=0.002. Among the covariates, LDLc was a predictor of GO clinical response at W6 (p=0.006), again LDLc and HoH were found to be significant independent predictors of the clinical response of GO to ivGCs at W12 (p=0.05 and p=0.04, respectively). Specifically, for each unit increase in LDLc, the odds of being classified as 'improved' at W12 decreased by a factor of 1.01 (odds ratio (OR) 0.989, 95% CI 0.979-1). When keeping all the other independent variables constant, patients with a HoH were 2.9 times less likely to be classified as 'improved' at W12 than patients without a HoH (OR 0.344, 95% CI 0.124-0.951).

A further multivariate model aimed at predicting W24 outcome was found to be significant (χ^2^(2)=9.458, p=0.009). LDLc and HoH were found to be almost related with the clinical outcome to ivGCs (p=0.09 and p=0.052, respectively) (**Tables 6A–C**).

**Table 6A:** Multivariate regression analysis: outcome at W6.

	B	Wald	P	OR (95% CI)
**LDLc**	-0.017	7.584	0.006	0.98 (0.97-0.99)
**HoH**	-0.381	0.67	0.413	0.68 (0.27-1.7)
**Costant**	2.045	6.93	0.008	7.73

**Table 6B:** Multivariate regression analysis: outcome at W12.

	B	Wald	P	OR (95% CI)
**LDLc**	-0.011	3.825	0.05	0.99 (0.98-1)
**HoH**	-1.068	4.228	0.04	0.34 (0.12-0.95)
**Age**	-0.013	0.74	0.39	0.99 (0.96-1.01)
**Costant**	1.896	4.165	0.041	6.66

**Table 6C:** Multivariate regression analysis: outcome at W24.

	B	Wald	P	OR (95% CI)
**LDLc**	-0.013	2.881	0.09	0.99 (0.97-1.01)
**HoH**	-1563	3773	0.052	0.21 (0.04-1.01)
**Costant**	1352	2	0.157	3.87

### Evaluation of GO clinical outcomes with a focus on specific components of the composite index (CI)

We finally evaluated the outcome of specific components of CI (Factor a: soft tissue involvement, Factor b: Hertel measurements, Factor c: presence of diplopia or ocular motility impairment, Factor d: eyelid aperture and Factor e: CAS) in relation to the OX-related variables studied. This analysis was not extended to W24 due to the small sample size for each component of the CI. As previously mentioned, patients were included into each analysis only if they started from a condition of "potential for improvement" (factor a: soft tissue involvement of almost grade A in at least one eye; factor b: Hertel ≥ 20 mm in at least one eye; factor c: diplopia or eyes motility involvement of almost one grade in at least one eye; factor d: eyelid aperture ≥ 13 mm in at least one eye; factor e: non exclusion criteria were applied because all patients had a baseline CAS ≥ 3). *Post hoc* analysis revealed that hypertension was negatively correlated with the parameters "increase in ocular motility of one grade or improvement in diplopia according to the Bahn–Gorman score". Among the 31 hypertensive patients evaluated at W12, only 4 (12.9%) showed improvement in the aforementioned parameter. In contrast, among the 93 non-hypertensive patients evaluated at W12, 32 (34.4%) experienced improvement, while 61 (65.6%) did not (OR=0.282, 95% CI=0.091-0.878, p=0.022). No statistically significant associations were found between hypertension and the following CI parameters: reduction in eyelid aperture ≥ 3 mm (p= 0.083, N. 27 patients enrolled), reduction in soft tissue involvement by at least 2 points (p=0.276, N. 135 patients enrolled), reduction in proptosis ≥ 2 mm (p=0.175, N. 107 patients enrolled), reduction in CAS ≥ 2 (p=0.218, N. 139 patients enrolled).

**Figure 1:**
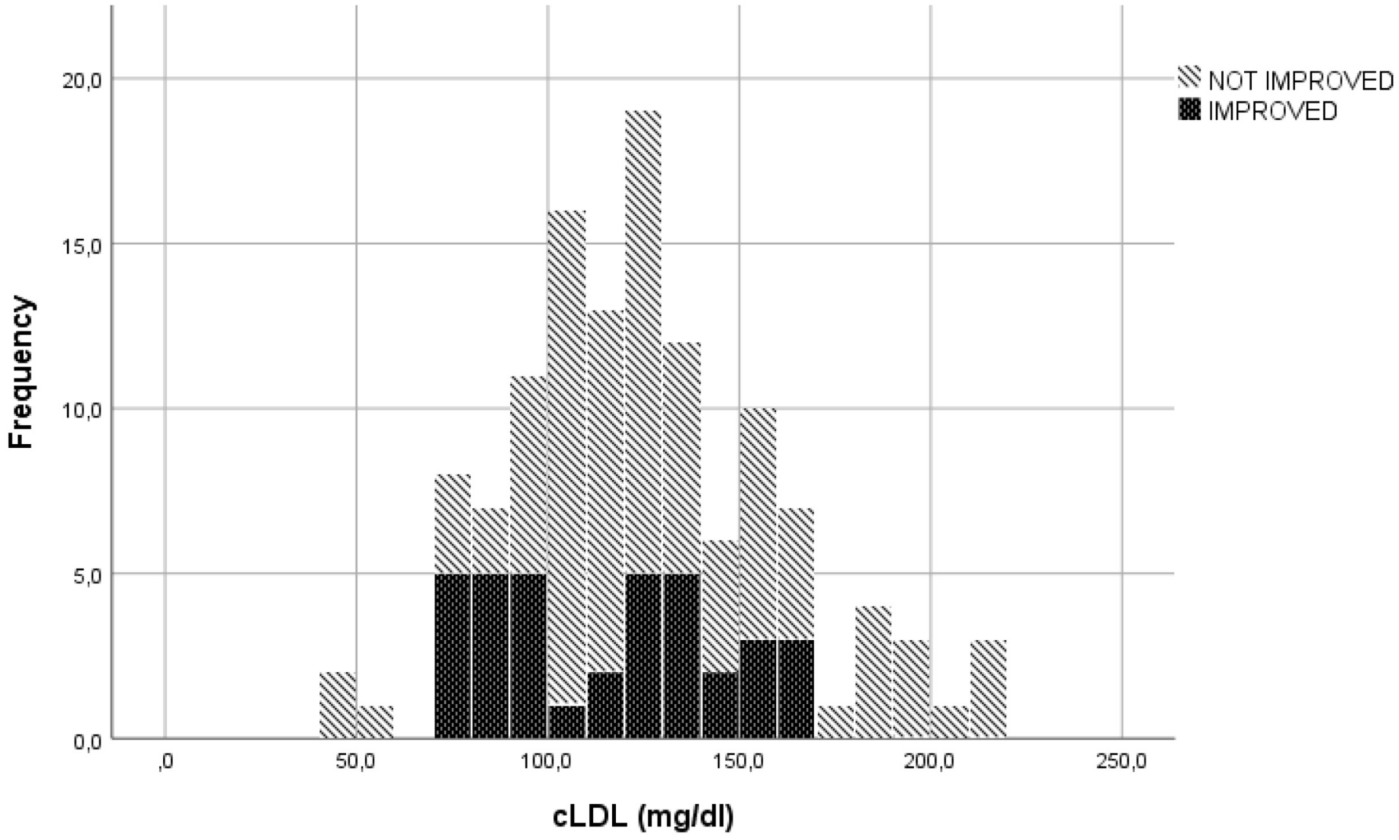
Eyes motility or diplopia improvement or non-improvement to ivGCs as function of LDLc levels.

**Figure 2:**
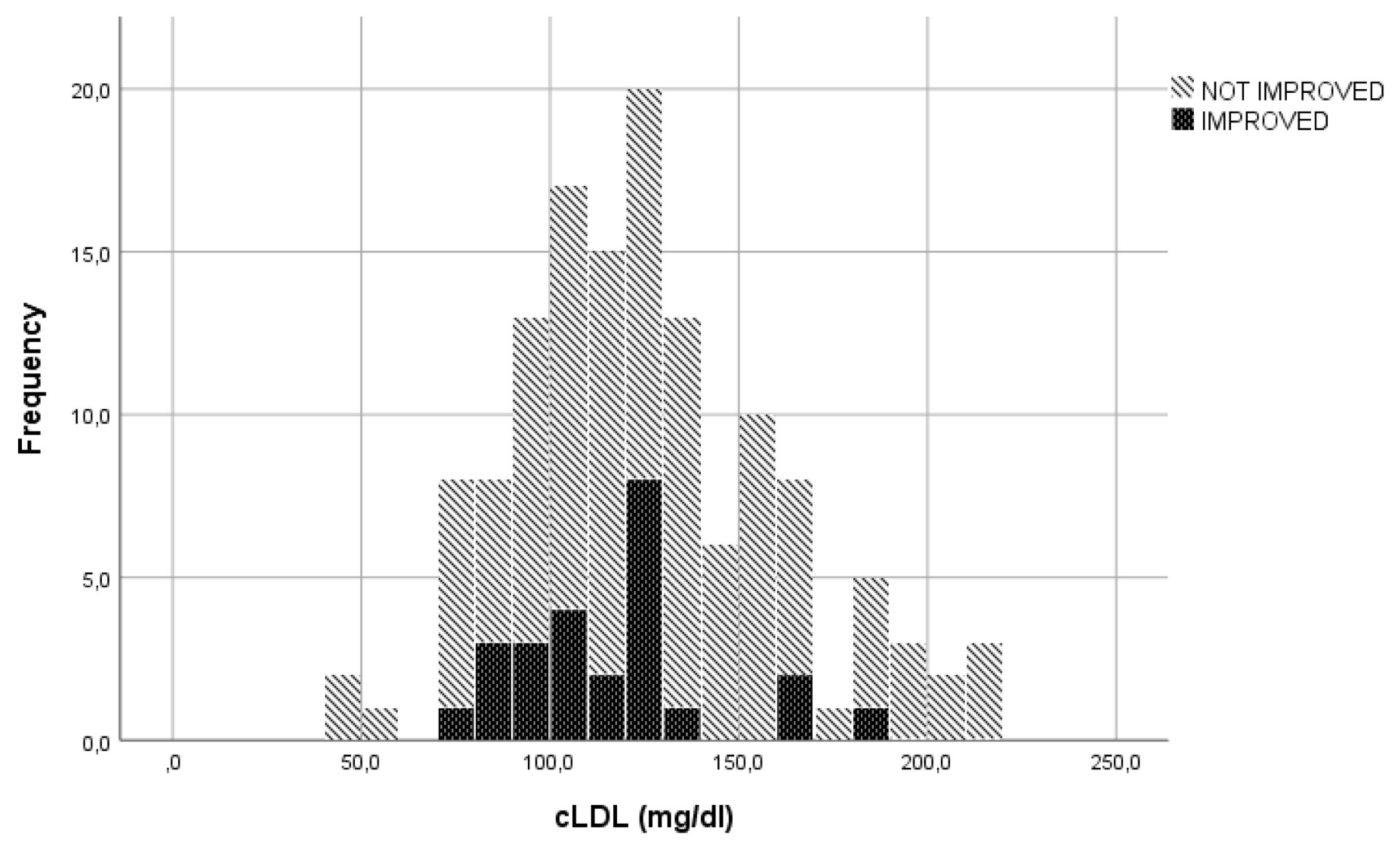
Soft tissues improvement or non-improvement to ivGCs as function of LDLc levels.

In the evaluation of LDLc, cut-offs used to transform the continuous variable into a categorical variable, were established through visual inspection of the graphs represented (**Figures 1**, **2**). The specific cut-offs values were chosen with reference to the Adult Treatment Panel III standard levels for LDLc (19). More specifically, the cut-offs ≥130 mg/dl ("bordeline high"), ≥175 mg/dl ("high") and ≥190 mg/dl ("very high") were adopted.

The LDLc blood concentration seemed to be correlated with the parameter "reduction in soft tissue involvement by at least 2 points". Of the 51 patients with LDLc levels > 130 mg/dl, 47 (92.2%) were classified as showing no clinical improvement in this parameter. Conversely, 63 (75%) of the 84 patients with LDLc concentrations < 130 mg/dl did not improve. (p=0.013).

Among the 112 patients with a serum LDLc concentration < 175 mg/dl, 36 (32.1%) showed clinical improvement at W12 in terms of an increase in ocular motility of almost one grade or improvement in diplopia according to the Bahn–Gorman score. However, none of the 12 patients with a serum LDLc concentration > 175 mg/dl were classified as improved (p=0.018).

Moreover, 45 (44.6%) out of the 101 patients with LDLc levels < 190 mg/dl showed improvement in the parameter 'reduction in proptosis ≥ 2 mm', whereas none of the 6 patients with LDLc values >190 mg/dl were classified as improved (p=0.039).

No significant difference was observed regarding the parameter "reduction in eyelid retraction ≥ 3 mm" when the LDLc cut-off was 130 mg/dl (p=0.662).

## Discussion

Our study was mainly focused on the putative effect of several clinical conditions related to oxidative stress on the short-, medium- and long-term clinical outcomes of active moderate-to-severe GO in patients receiving treatment with intravenous glucocorticoids.

In our retrospective cohort of patients with active moderate-severe GO receiving ivGCs, we were able to model the predicted probability of an individual patient being classified as "improved" at W6, W12, or W24 based on serum levels of LDLc at baseline. Additionally, hypertension reduced the patient's likelihood of being classified as "improved" at W12 by 3.5 times. Hypercholesterolemia promotes low-grade chronic inflammation caused by increased levels of oxLDLc and free fatty acids (FFA) (20), which induce systemic lipotoxicity and endoplasmic reticulum (ER) stress, a condition characterized by dysregulation of the synthesis, folding, repair and trafficking of several proteins (21). Lipotoxicity and ER stress are associated with the modulation of tissue macrophage functions, such as the synthesis of cytokines, chemotaxis, phagocytosis, proliferation, antigen presentation and the generation of oxidative species (ROS), which favor fibrogenic response activation by interleukin-6 (IL-6). Indeed, the increase in IL-6 activity in the intraorbital environment promotes the secretion of insulin-like growth Factor 1 (IGF-1) by intraorbital fibroblasts and stimulates fibroblast proliferation, with subsequent orbital soft tissue expansion through increased Insulin Growth Factor (IGF-1) and Thyroid Stimulating Hormone (TSH) receptors cross-talk (22). It also appears that IL-6 activates the JAK/STAT pathway modulating the activity of glucocorticoids receptors and consequently the effectiveness of pulse therapy in patients with GO (23–25). On the other hand, oxLDL regulates the expression of dipeptidyl dipeptidase IV (DPP4) in macrophages, leading to an increase in CD36+ cells, which contributes to the inflammatory processes of atherosclerosis in obese and insulin-resistant patients (26). Furthermore, it has been suggested that LDLc has direct effects on the transcriptional and translational activity of corticosteroids at the cellular level and on hepatic mRNA levels (26–28).

The role of hypertension is likely based on its association with chronic oxidative stress and innate and acquired immunity, as supported by emerging evidence in the literature. Recent data have suggested that hypertension and decreased nitric oxide (NO) are associated with the increase in OX. OX contribute to the activation of the PI3K/Akt-MAPK pathway causing overexpression of the genes related to redox activity (29, 30). Elevated blood pressure (BP) can induce organ damage promoting the formation of new antigens which sustains the low-grade inflammation condition and trigger the activation of the innate immunity by activating the Toll-like receptors (TLRs) on antigen-presenting cells, moreover the new antigens enhance the immunogenicity of dendritic cells and facilitate the production of proinflammatory cytokines (31, 32).

Chronic hyperactivation of the renin-angiotensin-aldosterone system (RAAS) is typically found in patients suffering from high BP. An increase in RAAS activity in turn leads to an increase in serum aldosterone levels. Through the induction of numerous transcription factors involved in inflammation, such as nuclear factor-kB (NF-kB) and activator protein-1 (AP-1), aldosterone appears to be responsible for the activation of adhesion molecules, including intercellular adhesion molecule-1 (ICAM-1) and connective tissue growth factor (CTGF). These molecules have been shown to induce inflammation and fibrosis (33). Furthermore, the latter seems to favor the activation and infiltration of macrophages at the endothelial level, with subsequent deposition of collagen and release of factors promoting fibrosis, such as transforming growth factor β1 (TGF-β1). Hyperactivation of the renin-angiotensin-aldosterone system is considered a cause of increased OX and, consequently, a risk factor for worse clinical outcomes in terms of response to treatment (34, 35).

We also found that LDLc and HoH were two independent predictors of short- and medium-term clinical outcomes, respectively, of the specific components of the CI of GO that are characteristic of the clinical phenotype of GO. An LDLc ≥ 130 mg/dl (19) was associated with an impaired response of the soft tissues of the orbit to glucocorticoids. Furthermore, LDLc values ≥ 175 mg/dl and ≥ 190 mg/dl reduced the clinical outcomes of ocular motility and proptosis to ivGCs. HoH reduced the odds of improvement in ocular motility (p=0.02).

It is not surprising that a slightly elevated LDLc level (>130 mg/dl) was associated with a weak response of eyelid oedema to glucocorticoids. Soft tissues of the orbit are usually the first structures involved in the immune-inflammatory process of GO characterized by sparse mononuclear cells infiltrate within the muscle endomysium and the fatty connective tissue, the overproduction of glycosaminoglycans is considered the pathogenetic cause of their remodeling (36, 37). The production of glycosaminoglycans by activated orbital fibroblasts is the first step in the morphological changes of orbital soft tissues and is related to cytokine production derived mainly from the activity of macrophages (38), that is modulated by cholesterol flux (39).

Furthermore, the impairment of ocular motility and the worsening of proptosis are associated with fibrotic processes that are mainly related to the increase in oxidative stress caused by T lymphocytes and can be favored by high levels of LDLc (≥ 175 mg/dl), hypertension-related oxidative processes and the activation of intracellular pathways induced by TRAbs binding to the TSH and IGF-1 receptors (40–42).

In conclusion, LDLc and hypertension are clinical conditions related to oxidative stress that negatively modulate the response of active moderate to severe GO to parental glucocorticoids. Notably, slightly elevated LDLc levels (> 130 mg/dl) were sufficient to reduce the response of orbital soft tissue to glucocorticoids, whereas more elevated LDLc levels (from 175 mg/dl to 190 mg/dl) and hypertension were associated with poorer eye motility and proptosis.

## Data availability statement

The raw data supporting the conclusions of this article will be made available by the authors, without undue reservation.

## Ethics statement

The studies involving humans were approved by Garibaldi Nesima Hospital ethical committee. The studies were conducted in accordance with the local legislation and institutional requirements. Written informed consent for participation was not required from the participants or the participants' legal guardians/next of kin in accordance with the national legislation and institutional requirements.

## Author contributions

RL: Conceptualization, Data curation, Formal analysis, Funding acquisition, Investigation, Methodology, Project administration, Resources, Software, Supervision, Validation, Visualization, Writing – original draft, Writing – review & editing. AN: Conceptualization, Data curation, Formal analysis, Funding acquisition, Investigation, Methodology, Project administration, Resources, Software, Supervision, Validation, Visualization, Writing – original draft, Writing – review & editing. GC: Investigation, Writing – review & editing. TP: Investigation, Writing – review & editing. DT: Investigation, Writing – review & editing. GP: Investigation, Writing – review & editing. FF: Methodology, Validation, Writing – review & editing, Investigation, Resources. AB: Investigation, Resources, Validation, Writing – review & editing, Supervision.

## References

[1] Wagner LH Bradley EA Tooley AA Ren Y Rachmasari KN Stan MN. Thyroid eye disease or Graves’ orbitopathy: What name to use, and why it matters. Front Endocrinol (Lausanne). (2022) 13:1083886. doi: 10.3389/fendo.2022.1083886 36518254 PMC9742525

[2] Schuh A Ayvaz G Baldeschi L Baretić M Bechtold D Boschi A. Presentation of Graves’ orbitopathy within European Group On Graves’ Orbitopathy (EUGOGO) centres from 2012 to 2019 (PREGO III). Br J Ophthalmol. (2024) 108(2):294–300. doi: 10.1136/bjo-2022-322442 PMC10850632 36627174

[3] Oeverhaus M Winkler L Stähr K Daser A Bechrakis N Stöhr M. Influence of biological sex, age and smoking on Graves’ orbitopathy – a ten-year tertiary referral center analysis. Front Endocrinol (Lausanne). (2023) 14:1160172. doi: 10.3389/fendo.2023.1160172 37082130 PMC10110835

[4] Lanzolla G Marcocci C Marinò M. Oxidative stress in graves disease and graves orbitopathy. Eur Thyroid J. (2020) 9:40–50. doi: 10.1159/000509615 PMC7802440 33511084

[5] Le Moli R Muscia V Tumminia A Frittitta L Buscema M Palermo F. Type 2 diabetic patients with Graves’ disease have more frequent and severe Graves’ orbitopathy. Nutr Metab Cardiovasc Dis. (2015) 25:452–7. doi: 10.1016/j.numecd.2015.01.003 25746910

[6] Naselli A Moretti D Regalbuto C Arpi ML Lo Giudice F Frasca F. Evidence that baseline levels of low-density lipoproteins cholesterol affect the clinical response of graves’ Ophthalmopathy to parenteral corticosteroids. Front Endocrinol (Lausanne). (2020) 11:609895. doi: 10.3389/fendo.2020.609895 33414766 PMC7784376

[7] Kalmann R Mourits MP. Diabetes mellitus: A risk factor in patients with Graves’ orbitopathy. Br J Ophthalmol. (1999) 83:463–5. doi: 10.1136/bjo.83.4.463 PMC1723016 10434871

[8] Sabini E Mazzi B Profilo MA Mautone T Casini G Rocchi R. High serum cholesterol is a novel risk factor for graves’ Orbitopathy: results of a cross-sectional study. Thyroid. (2018) 28:386–94. doi: 10.1089/thy.2017.0430 29336220

[9] Stein JD Childers D Gupta S Talwar N Nan B Lee BJ. Risk factors for developing thyroid-associated ophthalmopathy among individuals with graves disease. JAMA Ophthalmol. (2015) 133:290–6. doi: 10.1001/jamaophthalmol.2014.5103 PMC4495733 25502604

[10] Lanzolla G Sabini E Leo M Menconi F Rocchi R Sframeli A. Statins for Graves’ orbitopathy (STAGO): a phase 2, open-label, adaptive, single centre, randomised clinical trial. Lancet Diabetes Endocrinol. (2021) 9:733–42. doi: 10.1016/S2213-8587(21)00238-2 34592164

[11] Bartalena L Baldeschi L Boboridis K Eckstein A Kahaly GJ Marcocci C. The 2016 european thyroid association/european group on graves’ Orbitopathy guidelines for the management of graves’ Orbitopathy. Eur Thyroid J. (2016) 5:9–26. doi: 10.1159/000443828 27099835 PMC4836120

[12] Mourits MP Prummel MF Wiersinga WM Koornneef L. Clinical activity score as a guide in the management of patients with Graves’ ophthalmopathy. Clin Endocrinol (Oxf). (1997) 47:9–14. doi: 10.1046/j.1365-2265.1997.2331047.x 9302365

[13] Martin SS Giugliano RP Murphy SA Wasserman SM Stein EA Češka R. Comparison of low-density lipoprotein cholesterol assessment by Martin/Hopkins estimation, friedewald estimation, and preparative ultracentrifugation insights from the FOURIER trial. JAMA Cardiol. (2018) 3:749–53. doi: 10.1001/jamacardio.2018.1533 PMC6143070 29898218

[14] Shabana W Peeters E De Maeseneer M. Measuring thyroid gland volume: Should we change the correction factor? Am J Roentgenol. (2006) 186:234–6. doi: 10.2214/AJR.04.0816 16357408

[15] Rosenthal R. Meta-Analytic T Procedures Fob Social Research. (Newbury Park, CA: Rosental publications) (1991). doi: 10.4135/9781412984997

[16] Box GEP Tidwell PW. Transformation of the independent variables. Technometrics. (1962) 4:531–50. doi: 10.1080/00401706.1962.10490038

[17] Nagelkerke NJD. A note on a general definition of the coefficient of determination. Biometrika. (1991) 78:691–2. doi: 10.1093/biomet/78.3.691

[18] Menard S. *Applied logistic regression analysis. Sage University Paper Series on Quantitative Applications in the Social Sciences.*, *07*–*106* . Thousand Oaks: Sage Publications, Inc. (1995).

[19] National Cholesterol Education Program (NCEP). Executive summary of the Third Report of the National Cholesterol Education Programme Vol. 285. NCEP (2001). 285:2486–97. doi: 10.1001/jama.285.19.2486 11368702

[20] Le Moli R Vella V Tumino D Piticchio T Naselli A Belfiore A. Inflammasome activation as a link between obesity and thyroid disorders: Implications for an integrated clinical management. Front Endocrinol (Lausanne). (2022) 13:959276. doi: 10.3389/fendo.2022.959276 36060941 PMC9437482

[21] Kushwaha RS McMahan CA Mott GE Carey KD Reardon CA Getz GS. Influence of dietary lipids on hepatic mRNA levels of proteins regulating plasma lipoproteins in baboons with high and low levels of large high density lipoproteins. J Lipid Res. (1991) 32:1929–40. doi: 10.1016/S0022-2275(20)41896-6 1687745

[22] Lanzolla G Maglionico MN Comi S Menconi F Piaggi P Posarelli C. Sirolimus as a second-line treatment for Graves’ orbitopathy. J Endocrinol Invest. (2022) 45:2171–80. doi: 10.1007/s40618-022-01862-y PMC9525329 35831587

[23] Johnson DE O’Keefe RA Grandis JR. Targeting the IL-6/JAK/STAT3 signalling axis in cancer. Nat Rev Clin Oncol. (2018) 15:234–48. doi: 10.1038/nrclinonc.2018.8 PMC5858971 29405201

[24] Hoki T Vladan P Olivera M-A Beleslin-HokiT BB MarkoviT D BuaI M. Proinflammatory cytokine IL-6 and JAK-STAT signaling pathway in myeloproliferative neoplasms. Mediators Inflammation. (2015). doi: 10.1155/2015/453020 PMC4602333 26491227

[25] Meyer LK Verbist KC Albeituni S Scull BP Bassett RC Stroh AN. JAK/STAT pathway inhibition sensitizes CD8 T cells to dexamethasone-induced apoptosis in hyperinflammation. Blood. (2020) 136:657–68. doi: 10.1182/blood.2020006075 PMC7414590 32530039

[26] Rao X Zhao S Braunstein Z Mao H Razavi M Duan L. Oxidized LDL upregulates macrophage DPP4 expression via TLR4/TRIF/CD36 pathways. EBioMedicine. (2019) 41:50–61. doi: 10.1016/j.ebiom.2019.01.065 30738832 PMC6441950

[27] Baschant U Tuckermann J. The role of the glucocorticoid receptor in inflammation and immunity. J Steroid Biochem Mol Biol. (2010) 120:69–75. doi: 10.1016/j.jsbmb.2010.03.058 20346397

[28] Enuka Y Feldman ME Chowdhury A Srivastava S Lindzen M Sas-Chen A. Epigenetic mechanisms underlie the crosstalk between growth factors and a steroid hormone. Nucleic Acids Res. (2017) 45:12681–99. doi: 10.1093/nar/gkx865 PMC5727445 29036586

[29] Touyz RM. Reactive oxygen species, vascular oxidative stress, and redox signaling in hypertension: What is the clinical significance? Hypertension. (2004) 44:248–52. doi: 10.1161/01.HYP.0000138070.47616.9d 15262903

[30] Cai H Harrison DG. Endothelial dysfunction in cardiovascular diseases: The role of oxidant stress. Circ Res. (2000) 87:840–4. doi: 10.1161/01.RES.87.10.840 11073878

[31] Norlander AE Madhur MS Harrison DG. Correction: The immunology of hypertension. J Exp Med. (2018) 215:719. doi: 10.1084/jem.2017177301022018c 29305396 PMC5789420

[32] Rizzoni D De Ciuceis C Szczepaniak P Paradis P Schiffrin EL Guzik TJ. Immune system and microvascular remodeling in humans. Hypertension. (2022) 79:691–705. doi: 10.1161/HYPERTENSIONAHA.121.17955 35098718

[33] Ekholm M Kahan T. The impact of the renin-angiotensin-aldosterone system on inflammation, coagulation, and atherothrombotic complications, and to aggravated COVID-19. Front Pharmacol. (2021) 12:640185. doi: 10.3389/fphar.2021.640185 34220496 PMC8245685

[34] Terada Y Ueda S Hamada K Shimamura Y Ogata K Inoue K. Aldosterone stimulates nuclear factor-kappa B activity and transcription of intercellular adhesion molecule-1 and connective tissue growth factor in rat mesangial cells via serum- and glucocorticoid-inducible protein kinase-1. Clin Exp Nephrol. (2012) 16:81–8. doi: 10.1007/s10157-011-0498-x 22042038

[35] Kotlyar E Vita JA Winter MR Awtry EH Siwik DA Keaney JF. The relationship between aldosterone, oxidative stress, and inflammation in chronic, stable human heart failure. J Card Fail. (2006) 12:122–7. doi: 10.1016/j.cardfail.2005.08.005 16520260

[36] Heufelder AE Bahn RS. Elevated expression in *situ* of selectin and immunoglobulin superfamily type adhesion molecules in retroocular connective tissues from patients with Graves’ ophthalmopathy. Clin Exp Immunol. (1993) 91:381–9. doi: 10.1111/j.1365-2249.1993.tb05913.x PMC1554712 7680294

[37] Hai YP Lee ACH Frommer L Diana T Kahaly GJ. Immunohistochemical analysis of human orbital tissue in Graves’ orbitopathy. J Endocrinol Invest. (2020) 43:123–37. doi: 10.1007/s40618-019-01116-4 31538314

[38] Smith TJ Wang HS Evans CH. Leukoregulin is a potent inducer of hyaluronan synthesis in cultured human orbital fibroblasts. Am J Physiol - Cell Physiol. (1995) 268. doi: 10.1152/ajpcell.1995.268.2.C382 7864077

[39] Choi HY Ruel I Choi S Genest J. New strategies to promote macrophage cholesterol efflux. Front Cardiovasc Med. (2021) 8:795868. doi: 10.3389/fcvm.2021.795868 35004908 PMC8733154

[40] Zhang L Grennan-Jones F Draman MS Lane C Morris D Dayan CM. Possible targets for nonimmunosuppressive therapy of graves’ orbitopathy. J Clin Endocrinol Metab. (2014) 99:1183–90. doi: 10.1210/jc.2013-4182 24758182

[41] Kahaly GJ Shimony O Gellman YN Lytton SD Eshkar-Sebban L Rosenblum N. Regulatory T-cells in Graves’ orbitopathy: Baseline findings and immunomodulation by anti-T lymphocyte globulin. J Clin Endocrinol Metab. (2011) 96:422–9. doi: 10.1210/jc.2010-1424 21147887

[42] Raychaudhuri N Fernando R Smith TJ. Thyrotropin regulates IL-6 expression in CD34+ Fibrocytes: clear delineation of its cAMP-independent actions. PloS One. (2013) 8:1–2. doi: 10.1371/journal.pone.0075100 PMC3783445 24086448

